# Transcriptome analysis of *Populus* × *canadensis* ‘Zhongliao1’ in response to low temperature stress

**DOI:** 10.1186/s12864-023-09187-7

**Published:** 2023-02-17

**Authors:** Chengchao Yang, Xiaoyu Li, Yan Zhang, Hua Jin

**Affiliations:** 1 Liaoning Provincial Institute of Poplar, 115213 Gaizhou, China; 2 College of Environment and Bioresources, Dalian Minzu University, 116600 Dalian, China

**Keywords:** Poplar, Freeze–thaw injury, Overwintering, Low temperature stress, Cold stress response

## Abstract

**Background:**

Low temperatures are known to limit the growth and geographical distribution of poplars. Although some transcriptomic studies have been conducted to explore the response of poplar leaves to cold stress, only a few have comprehensively analyzed the effects of low temperature on the transcriptome of poplars and identified genes related to cold stress response and repair of freeze–thaw injury.

**Results:**

We exposed the Euramerican poplar Zhongliao1 to low temperatures; after stems were exposed to − 40℃, 4℃, and 20℃, the mixture of phloem and cambium was collected for transcriptome sequencing and bioinformatics analysis. A total of 29,060 genes were detected, including 28,739 known genes and 321 novel genes. Several differentially expressed genes (n = 36) were found to be involved in the Ca^2+^ signaling pathway, starch–sucrose metabolism pathway, abscisic acid signaling pathway, and DNA repair. They were functionally annotated; glucan endo-1,3-beta-glucosidase and UDP-glucuronosyltransferase genes, for instance, showed a close relationship with cold resistance. The expression of 11 differentially expressed genes was verified by qRT-PCR; RNA-Seq and qRT-PCR data were found to be consistent, which validated the robustness of our RNA-Seq findings. Finally, multiple sequence alignment and evolutionary analysis were performed, the results of which suggested a close association between several novel genes and cold resistance in Zhongliao1.

**Conclusion:**

We believe that the cold resistance and freeze–thaw injury repair genes identified in this study are of great significance for cold tolerance breeding.

## Background

Temperature is the main limiting factor that determines the geographical distribution of plants. The global economic losses caused upon exposure of plants to low temperatures are substantial each year. Yang et al. [1] investigated the mechanism underlying overwintering death in poplar; in regions such as Liaoning Province of China, overwintering death can be attributed to the accumulation of freeze–thaw damage beyond the limits of freeze–thaw resistance.

On exposure to low temperature stress, the response of plants involves several steps, including signal perception, transduction, and amplification, involving a series of primary and secondary messengers. The mechanisms of cold signal perception and transduction have been widely explored. It is generally believed that the cell membrane first senses changes in ambient temperature [2] and then transmits the signal to the cell. Protein kinase, Ca^2+^ channels, and phospholipase appear to play the role of secondary temperature receptors [3–6]. The phytochrome phyB and phototropin2 not only act as photoreceptors but also can sense changes in ambient temperature [7, 8].

There have been no recent breakthroughs in research on the antifreeze mechanism of plants. Damage to the cell membrane structure and oxidative damage caused by reactive oxygen species are known to be largely involved. In poplars, freeze–thaw injury can be classified into reversible and irreversible freeze–thaw injury. The cumulative effect of effective freeze–thaw injury is the main cause of poplar death during overwintering [1, 9]. To ensure survival during harsh winters, the ability to recover from reversible freeze–thaw injury is essential. At present, most studies have focused on elucidating the molecular mechanisms underlying the response of plants to cold and subzero temperatures. For example, single-molecule real-time (SMRT) and Illumina RNA sequencing were performed for *P*. *ussuriensis* after chilling (3 °C) and freezing (− 3 °C) stresses [10]. Chen et al. [11] performed deep-sequencing transcriptome analyses of low temperature (4 °C and − 4 °C) perception in a desert tree. *P. euphratica*, providing a global transcriptome picture of *P. euphratica* under low temperature stress. Besides, Yang et al. [12] conducted transcriptome profiling of *P. tomentosa* leaves under cold stress(4 °C). In *P. euphratica*, abscisic acid(ABA) and Ca^2+^ signaling components play a key role in adaptation to cold stress [6], while in *P. tomentosa* [12], cold response candidate genes are mainly associated with glucose metabolism, antioxidant defense system, hormone signal transduction, and photosynthesis (e.g., genes encoding sucrose synthase, superoxide dismutase, brassinosteroid-signaling kinase, and ferredoxin). Wang et al. [13] performed high-throughput sequencing to generate global transcriptome profiles of *Pinus koraiensis* under cold stress(− 20 °C), enhancing our understanding of cold response-related molecular mechanisms and providing the basis for the molecular breeding of conifers. To date, however, not much is known regarding the molecular mechanisms used by poplars to recover from freeze–thaw injury.

Herein *Populus × euramericana* exposed to different temperatures was subjected to RNA-Seq; genes involved in freeze–thaw resistance were identified, and real-time PCR (qRT-PCR) verification and pathway analysis were conducted.

## Methods

### Poplar culture, collection, and processing

Zhongliao1 (*Populus* × *canadensis* ‘Zhongliao1’) obtained a superior seed certificate from Liaoning Province Forest Quality Examination and Approval Committee, certified seed code: Liao S-SC-PC-001-2011. Zhongliao1 was deposited in the National Center for Forestry and Grassland Genetic Resources (http://www.nfgrp.cn/data/list/resource_detaillist.html), platform resource number: 1111C0003911000007.

In late April, Zhongliao1 was cultured using the hardwood cutting method in a sandy loam nursery (N41°12′8.66′′, E121°24′1.20′′, altitude of 16.85 m) in Linghai City, Liaoning Province, China. In mid-November, 1-year-old lignified stems were collected, cut into 50-cm-long segments, and exposed to − 40 °C for 24 h, 4 °C for 24 h, and 20 °C for 24 h (45 stems per treatment pattern). The samples treated at − 40℃, 4 °C, and 20 °C were collected at 20 °C and the collection of samples treated at each temperature was completed within 10 min. Before sampling, the epidermis of the stem segment was removed, and cambium and phloem were scraped with a stainless-steel blade; the samples were then transferred into a cryopreservation tube, immediately frozen in liquid nitrogen, and stored at − 80℃ until needed.

### Determination of physiological parameters

Relative electrical conductivity (REC), malondialdehyde (MDA), proline, and soluble sugar contents were measured as previously described by Li et al. [14].

### RNA extraction, cDNA library construction, and RNA-Seq

The samples were ground into a fine powder in liquid nitrogen. RNA was extracted using TRIzol (Invitrogen, USA), according to manufacturer instructions. RNA concentration, RIN value, 28 S/18S ratio, and fragment size of total RNA were measured using Agilent RNA 6000 Nano Kit on an Agilent 2100 Bioanalyzer. RNA sample purity was determined by measuring absorbance with a NanoDrop™ spectrophotometer.

mRNA was isolated from total RNA using magnetic beads with oligo(dT). To fragment mRNA, an appropriate amount of interrupting reagent was added to the obtained mRNA sample under high temperature; this fragmented mRNA was then used as a template to synthesize double-stranded cDNA, which was purified using a kit. The cohesive ends were repaired and the base “A” was added to the 3′-end of cDNA to ligate the adaptor. Fragment sizes were then selected and amplified by PCR. The constructed library was subsequently analyzed by an Agilent 2100 Bioanalyzer (Agilent DNA 1000 Reagents) and ABI StepOnePlus Real-Time PCR System (TaqMan Probe). On passing the test, DNA was sequenced on an Illumina HiSeq 3000 platform by Beijing Genomics Institute (BGI), China.

### Bioinformatics analysis

SOAPnuke v1.5.2 was used to filter sequencing data [15]. The methods applied were as follows: (1) reads containing sequencing adaptors were eliminated; (2) reads with base ratio (base mass ≤ 5) > 20% were removed; (3) reads with unknown base (Nbase) ratio > 5% were deleted. The filtered clean reads were then stored in FASTQ format. The reads were subsequently mapped to a reference genome (https://phytozome.jgi.doe.gov/pz/portal.html#!info?alias=Org_Ptrichocarpa) using HISAT2 (v2.0.4) [16]. Bowtie2 v2.2.5 [17] was used to align the clean reads to the reference coding gene set, and gene expression levels were calculated by RSEM v1.2.12 [18]. Based on gene expression levels in different samples, a heatmap was drawn using pheatmap (v1.0.8). Differential expression analyses were performed with DESeq2(v1.4.5) with Q value ≤ 0.05. To further understand physiological changes, gene ontology (GO)(http://www.geneontology.org/) and Kyoto encyclopedia of genes and genomes (KEGG) (https://www.kegg.jp/) pathway enrichment analyses [19–21] were performed with differentially expressed genes (DEGs) using the R “Phyper” function (https://en.wikipedia.org/wiki/Hypergeometric_Distribution) based on the hypergeometric test. The significance levels of terms and pathways were assessed with Q value ≤ 0.05 via Bonferroni correction [22].

### qRT-PCR verification

RNA was extracted from the aforementioned samples and reverse transcribed into cDNA. Eleven DEGs were randomly selected for data verification by qRT-PCR.

qRT-PCR was performed with SYBR Premix Ex Taq II (Takara) on a StepOnePlus Applied Biosystems Real-Time Instrument (Thermo Fisher Scientific, USA). The cycling conditions were as follows: denaturation at 95 °C for 3 min, followed by 40 cycles of 95 °C for 10 s, 60 °C for 20 s, and 72 °C for 10 s, and final extension at 72 °C for 3 min. Table 1 shows the primers used for qRT-PCR. GAPDH served as the internal control. Three independent replicates were assessed. Relative gene expression was calculated using the 2^−ΔΔCt^ method.

**Table 1:** Primer pairs used for qRT-PCR

Sequence (5’-3’)	Gene name	Size
TAGGCTGTTGGAAAGGTTCTGCCA	GAPDH	171
TGCCTTCTTCTCAAGCCTGACAGT		
GTTGTGGTTGCCAATATCCCTA	Potri.013G154000. v3.0	86
CCATCTTCATTACCTTCCCTGT		
TAGCAGTGGCGTGACAGCAGAA	Potri.T011400.v3.0	82
TTGACATGGAAGACCAATAGAG		
AATGGATTCTATCTGTGCTGGT	Potri.001G313000.v3.0	134
CTTCGTCTCTTTTCACGTTGTT		
TTAGATAAAAGGTCGACGCGG	BGI_novel_G000247	119
CGAAAGTTGATAGGGCAGAAA		
TCAAAGAATCATCGAAGAAGGA	Potri.011G113000.v3.0	128
GCTGTAGAACGGAATAAGTGCC		
ATATGTCCTGCTTATGGTCTA	Potri.T079300.v3.0	135
CATCACTACTGTGAATTCTTC		
GGGGTTCGAGCCCTGGTGTTA	Potri.019G003400.v3.0	127
AAATGGGGGTGAGCTTGTGAG		
ATCAAGCCATATTATCCGCAA	Potri.008G055900.v3.0	97
GAACGAGACGAGAAATCCACT		
ACACAAAGGGCTAATAACCAG	Potri.001G036900.v3.0	103
GAACATAGGCAACACACACAA		
AGTGGAACTAAACCCAGGAAA	Potri.002G019300.v3.0	99
TGAGCAGCCCAGACAACGATA		
ATCTGTTTCTTTCTATCCCGT	Potri.019G020700.v3.0	125
TGATTTCATTACCCTCTTTCA		

### Multiple sequence alignment and evolutionary analysis

Highly homologous sequences of different species were queried systematically against NCBI using BlastX, and amino acid sequences were aligned by ClusterX. A phylogenetic tree was constructed by MEGA7 with the neighbor joining method.

We confirm that all methods of experimental research and field studies, including the collection of plant material, were performed in accordance with relevant institutional and international guidelines and legislation.

## Results

### Assessing physiological indices

**Fig. 1:**
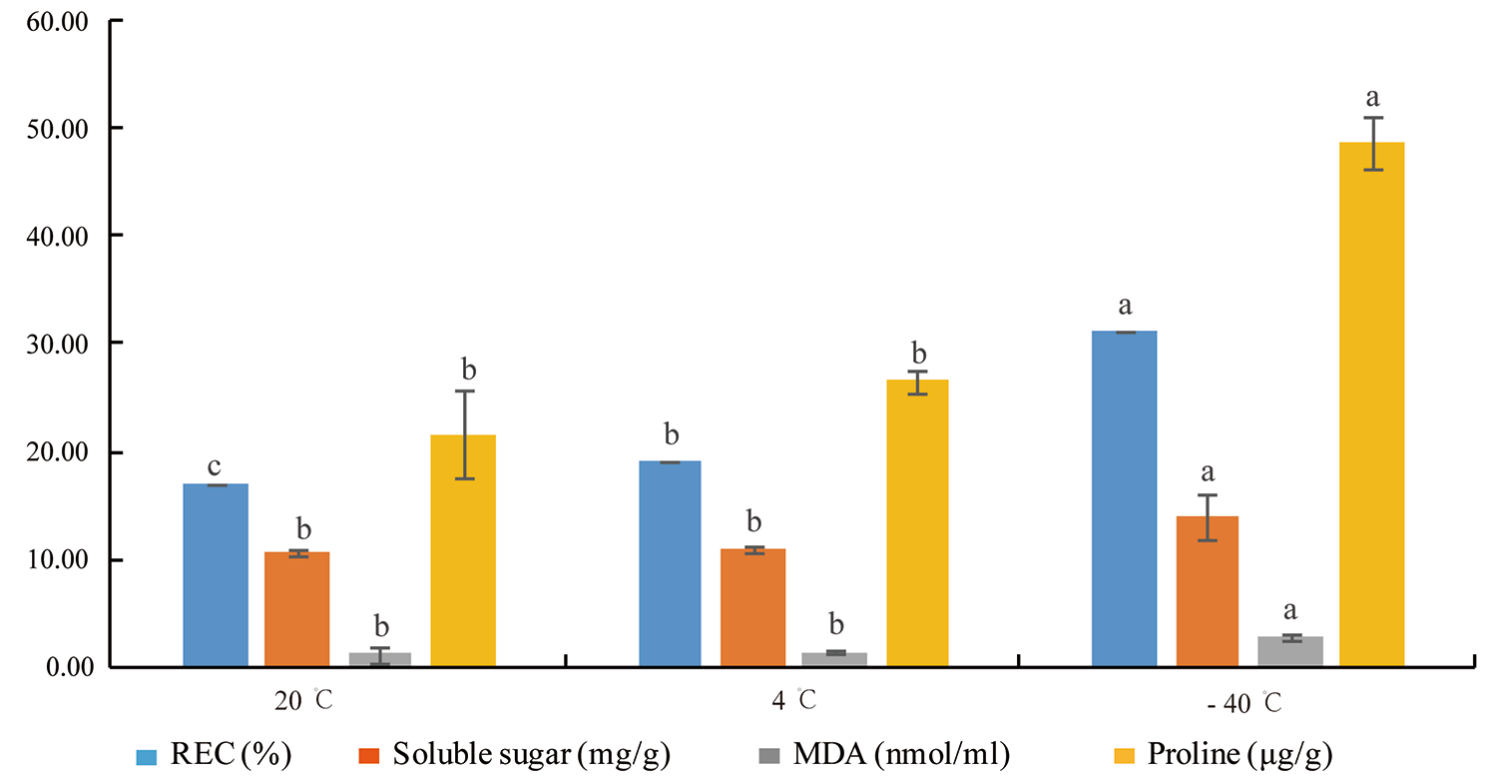
Effects of different temperatures on four physiological indices. Note: Letters on the columns of the same physiological index represent results of multiple comparisons. Different letters indicate that there was a significant difference between different temperatures, and same letters indicate that there was no significant difference

Electrolyte leakage and MDA, proline, and soluble sugar contents were measured. We found that proline, MDA, and soluble sugar contents slightly increased between 20 °C and 4 °C, but electrolyte leakage was significantly different (p < 0.01) at 20 °C and 4 °C. At − 40 °C, these physiological indices showed a significant increase; electrolyte leakage and proline content reached a very significant level (p < 0.01), while soluble sugar and MDA contents reached a significant level (p < 0.05) (Fig. 1). These findings indicated that with a decrease in temperature, the damage to the cell membrane increased; moreover, accumulation of membrane lipid peroxidation products was observed, resulting in gradual aggravation of cell membrane damage. Proline and soluble sugar contents were increased, indicating that the contents of osmotic adjustment were increased. This increase in osmotic potential seems to contribute to improving cold resistance.

### RNA-Seq

The collected poplar samples were treated at − 40 °C, 4 °C, and 20 °C. Each treatment was repeated thrice (n = 9 samples).

Six samples were assessed on the Illumina HiSeq platform, and each sample produced 6.71 Gb data on average. Further, on average, each sample generated 54.51 Mb raw reads and 44.72 Mb clean reads. The clean data were deposited in the NCBI Sequence Read Archive (SRA) under accession number PRJNA891633. One abnormal dataset was removed from each treatment, and the remaining two biological repetitions, i.e., data of six samples, were analyzed. Herein abnormal data indicate a group of data that could not be clustered with the other two groups of data in cluster analyses; i.e., it showed low correlation with the other two groups of data. The average comparison rate of the sample genome was 72.40%, and the average comparison rate of the gene set was 71.59%. A total of 29,060 genes were detected, including 28,739 known genes and 321 predicted novel genes (Table 2).

**Table 2:** Basic information and splicing analysis of RNA-Seq data

Sample	Total raw reads (Mb)	Total clean reads (Mb)	Total clean bases (Gb)	Clean reads Q20 (%)	Clean reads ratio (%)	Total mapping ratio	Total gene number	Known gene number	Novel gene number
CK1	52.58	44.41	6.66	98.27	84.46	69.51	23,621	23,413	208
CK3	55.77	45.27	6.79	98.50	81.18	72.19	23,411	23,194	217
T201	55.2	44.8	6.72	98.47	81.16	71.73	25,503	25,276	227
T203	54.17	44.66	6.7	98.51	82.44	74.5	25,247	25,025	222
TN402	55.16	44.69	6.7	98.34	81.01	71.62	23,057	22,856	201
TN403	54.17	44.47	6.67	98.37	82.09	69.97	23,829	23,612	217

HISAT2 was used to compare clean reads to the reference genome sequence. The average comparison rate of each sample was 72.40%.

### Identification of cold stress response genes

**Fig. 2:**
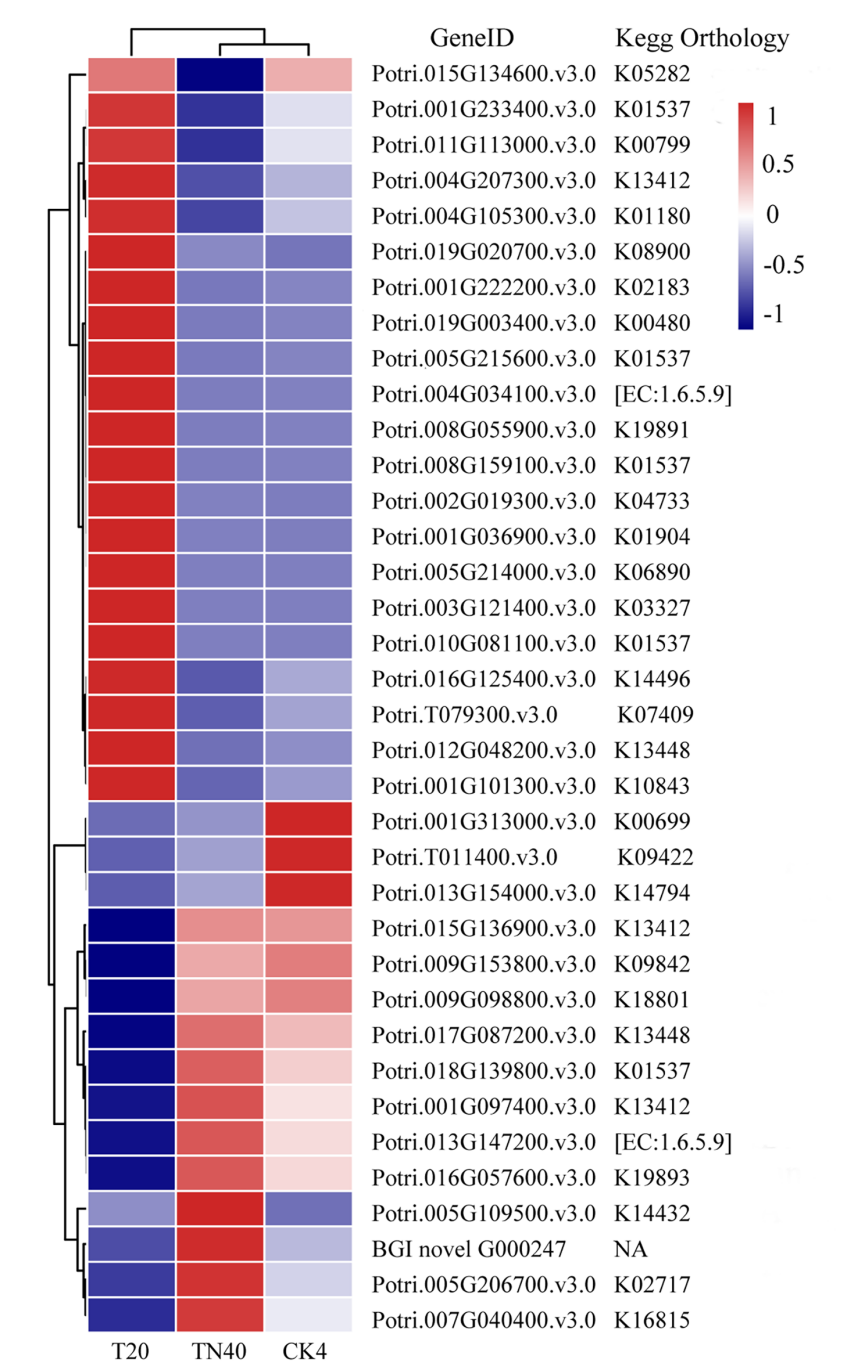
Heatmaps and annotations of 36 DEGs

Based on gene expression levels, we identified DEGs that were induced in response to cold stress (Table 3). There were 10,105 cold-induced genes, 2,910 cold-repressed genes, and 12,544 non-DEGs. Overall, 36 DEGs showed significantly up- or downregulated expression levels (Fig. 2).

**Table 3:** Statistical results of DEGs

	CK vs. T20	CK vs. TN40	T20 vs. TN40
Up Down	9606 2750	1097 1816	2910 10,105

### Verification of the reliability of RNA-Seq data by qRT-PCR

**Fig. 3:**
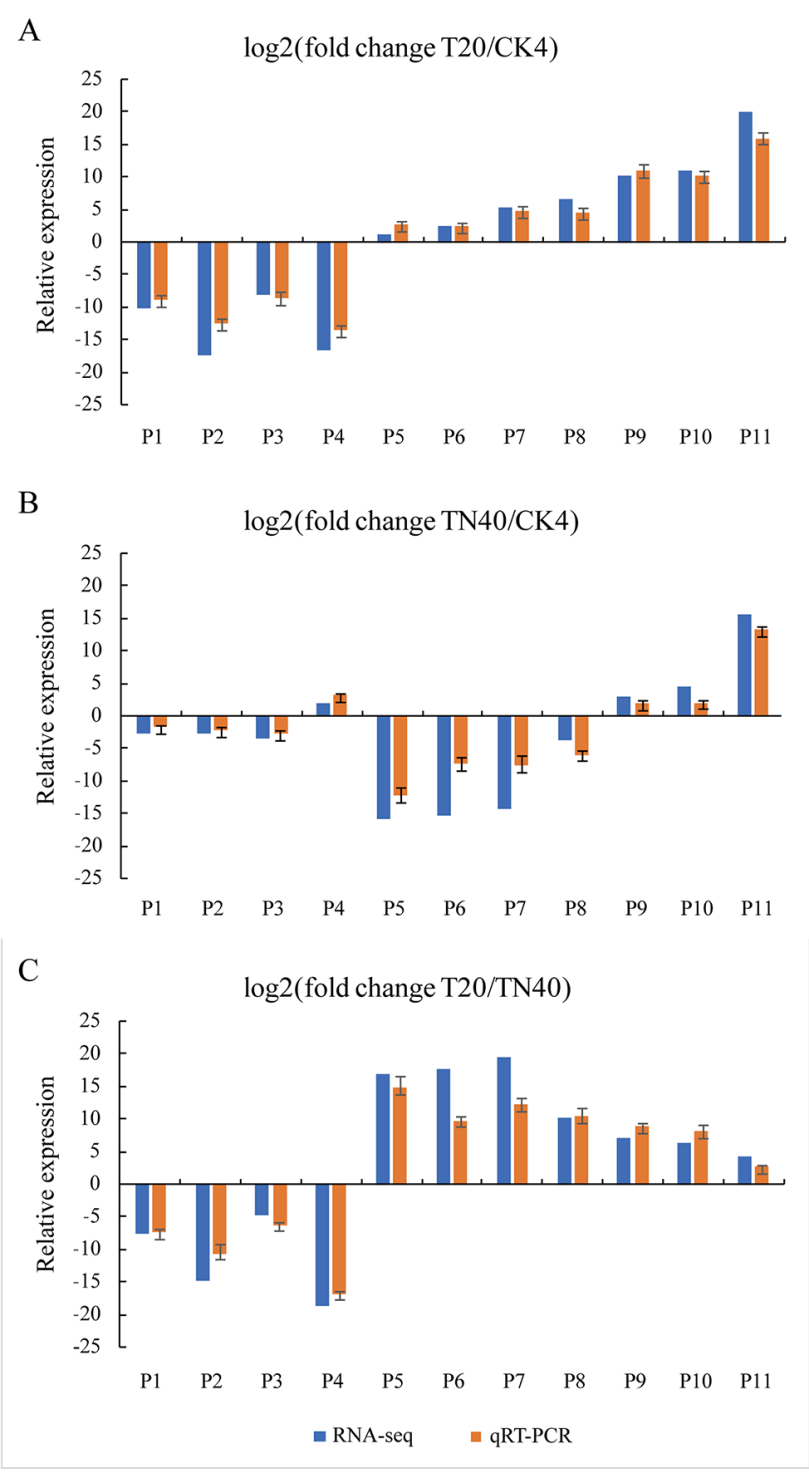
Comparison of changes in DEGs detected by RNA-Seq and qRT-PCR

To verify the accuracy of RNA-Seq data, we verified the expression levels of 11 randomly selected DEGs by qRT-PCR. A good correlation was found between RNA-Seq and qRT-PCR data (Fig. 3), validating the robustness of our RNA-Seq data.

### Functional annotation

#### GO annotation of cold stress response genes

**Fig. 4:**
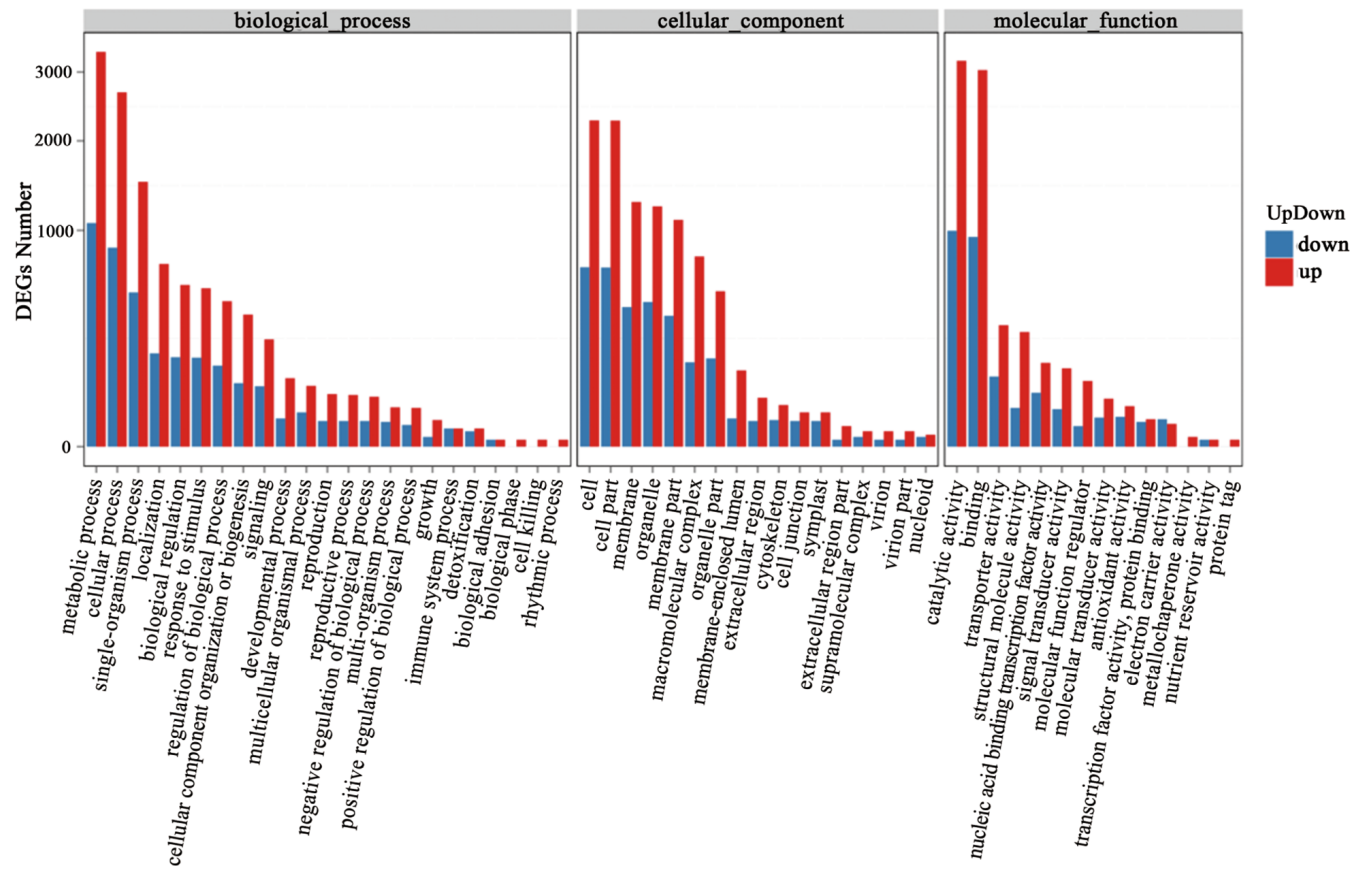
DEGs enriched in GO terms

All up- and downregulated genes were subjected to GO analysis (Fig. 4). DEGs were functionally classified into molecular function, cellular component, and biological process categories. The molecular function category mainly included 14 items, with catalytic activity, binding, transporter activity, structural molecule activity, nuclear acid binding transcription factor activity, and signal transducer activity being the top 6 items. In the cellular component category, cell, cell part, membrane, organelle, and membrane part were the top 5 items. Finally, the biological process category mainly included 23 items, such as metabolic process, cellular process, single organization process, localization, biological regulation, response to stimulus, and reproduction.

#### Pathway analysis of cold stress response genes

KEGG pathway classification results were divided into five categories, with a total of 19 items. The most notable features were transport and catabolism, signal transduction, membrane transport, translation, and replication and repair.

**Fig. 5:**
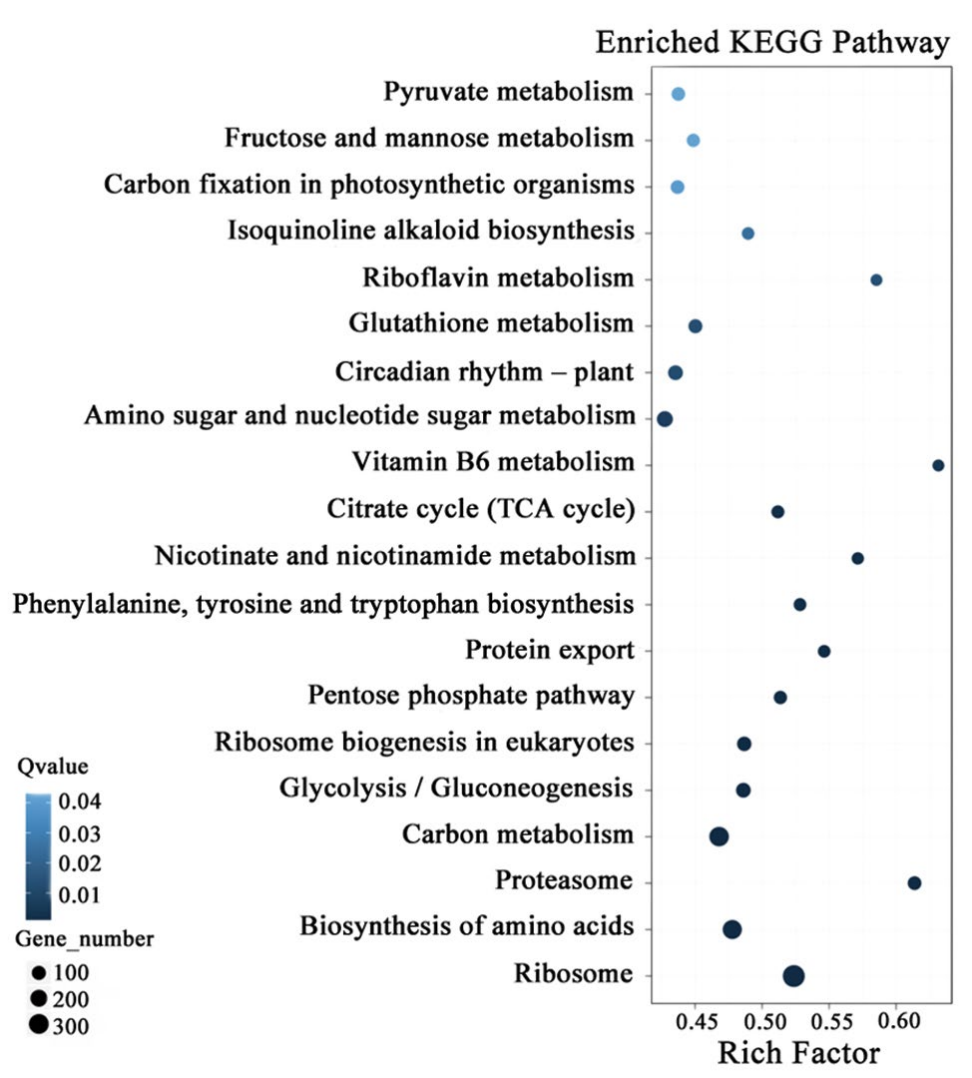
TN40-vs-T20 DEGs enriched in KEGG pathways

**Fig. 6:**
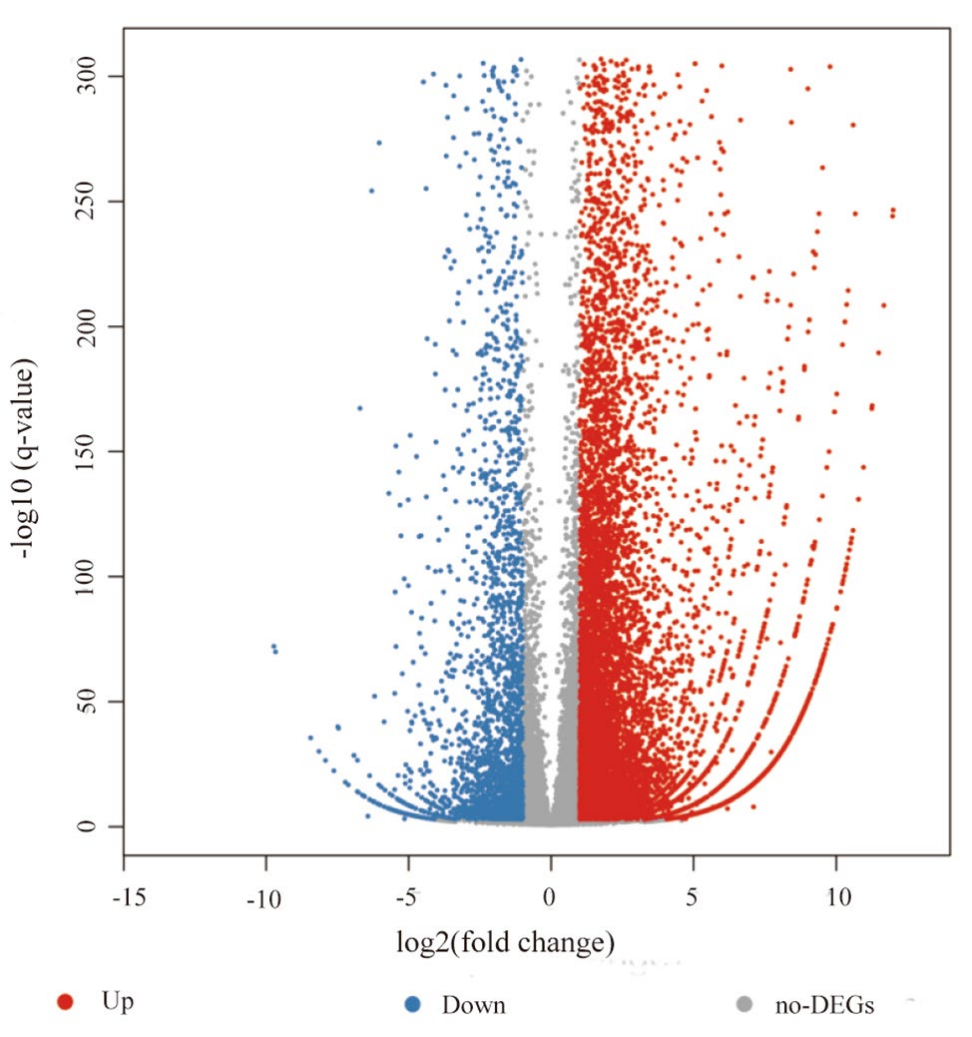
Volcano plot for TN40-vs-T20 DEGs. Notes: The *x*-axis shows log_2_-transformed fold-change, the *y*-axis shows − log_10_ transformed significance. Red points represent upregulated DEGs, blue points represent downregulated DEGs, and gray points represent non-DEGs.

Figure 5 shows the total functional enrichment results of DEGs of the top 20 KEGG pathways for TN40-vs-T20, and Fig. 6 shows a volcano plot of DEGs for TN40-vs-T20.

## Discussion

### Multiple sequence alignment and evolutionary analysis of new target genes

**Fig. 7:**
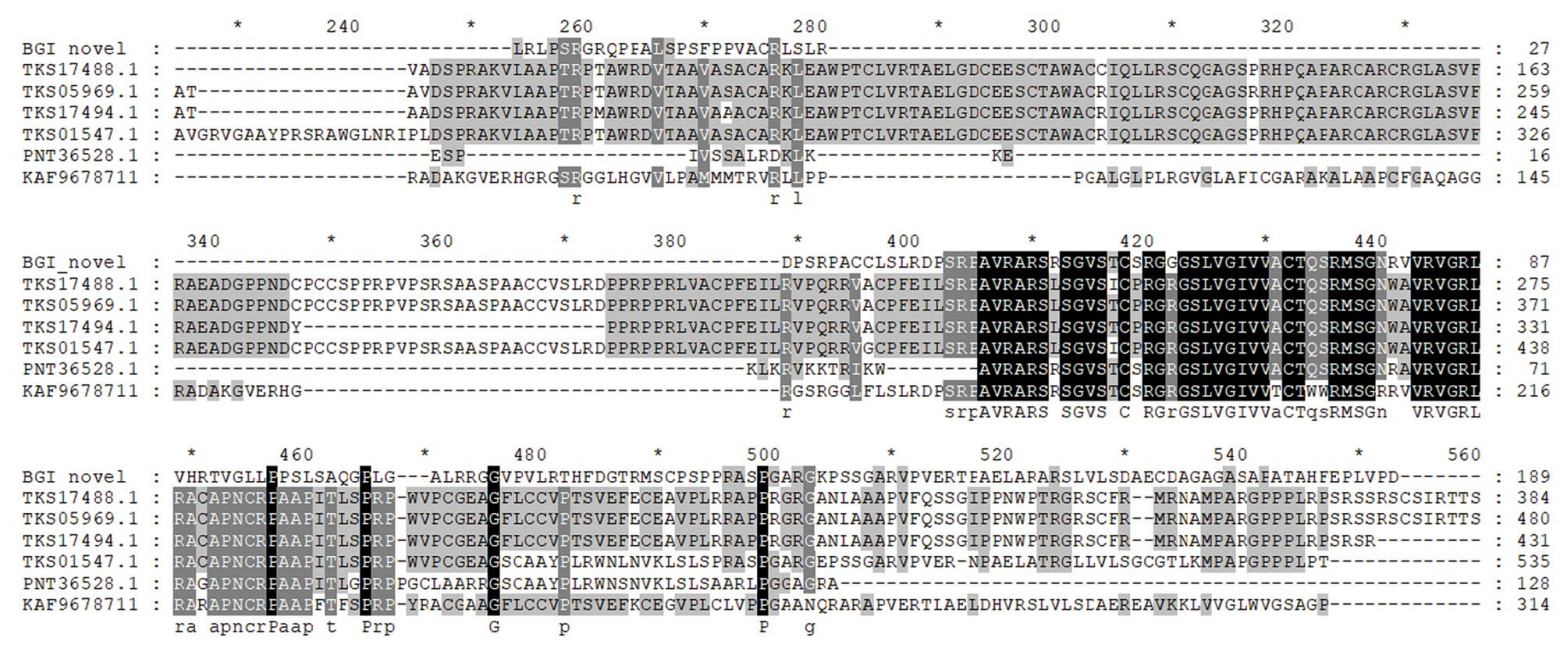
Comparison of amino acid multiple sequences

**Fig. 8:**
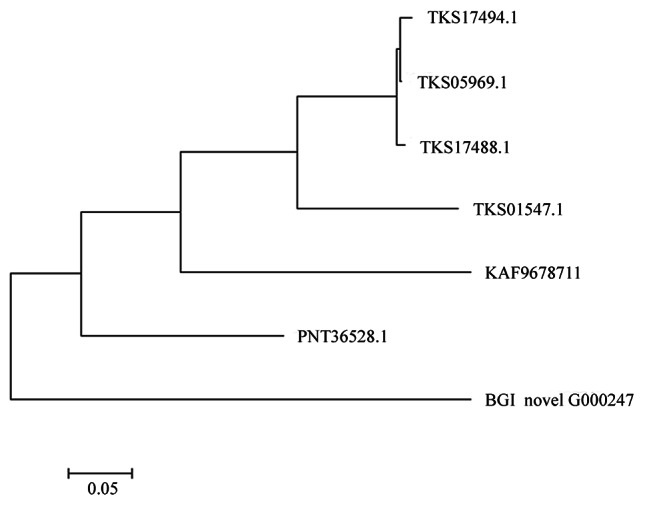
Evolutionary tree of seven genes

For novel gene mining, amino acid sequences of the novel gene BGIG00247 were used for BLAST analysis, and six genes with a relatively high similarity score from BLAST results were screened and further analyzed. A phylogenetic tree was constructed with MEGA7, and amino acid sequences of these seven genes were used for multiple sequence alignment (Fig. 7). BGIG00247 showed high similarities to the hypothetical protein POPTR005G132600 (PNT36528) from *P. trichocarpa* and KAF9678711 from *Salix dunnii*, suggestive of a close genetic relationship among them (Fig. 8). Another novel gene showed the highest BLAST score with a cold adaptation-related gene in silver poplars (TKS17494.1). Therefore, these novel genes seem to be closely associated with cold resistance in Zhongliao1.

### Relationship between DEGs and the Ca^2+^ signaling pathway

The cell membrane is the primary target of low temperature damage. Low temperatures cause the cell membrane to turn into the gel phase, leading to depolymerization and rearrangement of microtubules and microfilaments in the cell; in addition, the ion channels on the membrane are activated, which causes an instantaneous increase in cytosolic concentrations of Ca^2+^. As a second messenger, Ca^2+^ has been found to activate the low temperature signaling pathway, enabling plants to respond to low temperatures [23].

Intracellular Ca^2+^ ions play a key role in transducing cold signals by interacting with calmodulin (CAM), calcineurin B-like proteins, calcineurin B-like interacting protein kinases, and Ca^2+^-dependent protein kinases.

Low temperature response genes include those encoding Ca^2+^ ATPase, aspartate protease, CAM-like (CML) proteins, fructan exohydrolase, glucuronosyltransferase, and glyceraldehyde-3-phosphate dehydrogenase.

Of the 36 DEGs identified in this study, 12 were involved in Ca^2+^ signaling pathways [e.g., Ca^2+^-dependent protein kinases (K13412, EC2.7.11.1),Ca^2+^/CAM-dependent protein kinase II (K04515, EC2.7.11.17), Ca^2+^-binding protein CML (K13448), and P-type Ca^2+^ transporter (K01537, EC7.2.2.10)].

CAM (Ca^2+^-binding protein), an important component of the second messenger system, plays a key role in Ca^2+^ signal transduction. CML proteins are the main Ca^2+^ receptor in cells [24], serving as an intermediary in various cellular reactions mediated by Ca^2+^. CAM was highly induced at low temperatures.

Ca^2+^ ATPase homologs are found in all membrane systems, including plasma membrane, vacuolar membrane, endoplasmic reticulum membrane, mitochondrial membrane, and chloroplast envelope. Because Ca^2+^ acts as a second messenger in plant signal transduction, Ca^2+^ ATPase, as a membrane Ca^2+^ pump, plays a vital role in the transmission and amplification of extracellular environmental signals.

Ca^2+^-dependent protein kinases are a type of Ser/Thr protein kinases unique to plants. Their kinase activity is directly regulated by Ca^2+^ rather than by CAM. They play important roles in plant Ca^2+^ signal transduction and participate in the diverse physiological process, such as abiotic stress resistance.

Ca/CAM is the most conserved signal transduction cascade system in organisms, playing a regulatory role in various cell activities, such as stress response and cell proliferation.

Collectively, our findings provide insights into how poplars tackle low temperatures from a molecular biology perspective.

### Relationship between DEGs and the ABA signaling pathway

ABA, a stress response hormone, plays a key role in abiotic stress defense [25, 26]. PYR/PYL and RCAR family members represent the main receptors of ABA; on binding to ABA, their conformation changes [27]. Previous studies [28, 29] have reported that when plants encounter adverse environmental conditions, cellular ABA content increases. ABA binds to PYR/PYL/RCAR, and this ABA–PYR/PYL/RCAR complex then binds to PP2C to inhibit protein phosphatase activity. In its phosphorylated active state, SnRK2 activates the downstream transcription factor ABF/AREB, positively regulating the expression of ABA signal response genes [30–32] to improve plant cold resistance.

**Fig. 9:**
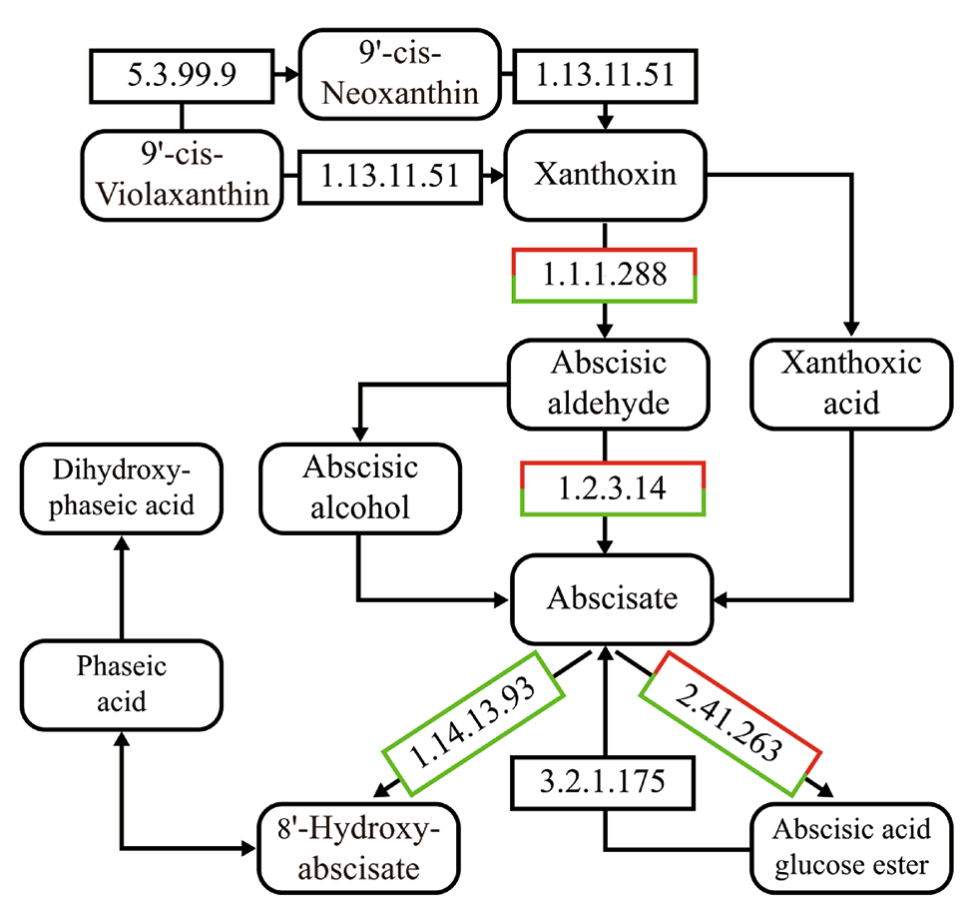
ABA biosynthesis pathway Note: Red indicates up- and green indicates downregulation

Herein three key genes in the ABA metabolic pathway (Fig. 9) were identified. First, the ABA signaling pathway-related K14496 (ABA receptor PYR/PYL family), which detects ABA signals and initiates the initial process of signal transduction. Second, the ABA responsive element binding factor K14432 (Potri.005G109500), is involved in the response to moderate low temperature stress and ABA signal transduction in poplars. Finally, the abscisic-aldehyde oxidase K09842 (EC1.2.3.14, Potri.009g153800) oxidizes ABA aldehyde to ABA, which represents the last and an important step in ABA biosynthesis [33].

ABA is a critical carotenoid-derived phytohormone; in recent years, its biosynthetic pathway in higher plants has been comprehensively investigated. ABA biosynthesis reportedly involves zeaxanthin epoxidase and 9-*cis*-epoxycarotenoid dioxygenase. ABA2 belongs to a cytosolic short-chain dehydrogenase/reductase that converts xanthoxin into abscisic aldehyde [33], which is an important step in the ABA synthesis pathway. The last step in this pathway is ABA aldehyde oxidation to ABA by abscisic-aldehyde oxidase.

### Relationship between DEGs and the starch–sucrose metabolism pathway

ABA and Ca^2+^ signaling components underlie a rapid, flexible cold response mechanism in *P. euphratica* [11]. Further, in *Camellia sinensis*, the carbohydrate metabolism and Ca^2+^ signaling pathways play a major role in cold stress response [34]. In this study, several key genes in the starch–sucrose metabolism pathway and the pentose and glucuronic acid conversion pathway were identified: endo-1,3(4)-beta-glucanase (K01180, EC3.2.1.6), glucan endo-1,3-beta-glucosidase 5/6 (K19893, EC3.2.1.39), glucan endo-1,3-beta-glucosidase 1/2/3 (K19891, EC3.2.1.39), glucuronoxylan 4-O-methyltransferase (K18801, EC2.1.1.112), cyanohydrin beta-glucosyltransferase (K13030, EC2.4.1.85), and glucuronosyltransferase (K00699, EC2.4.1.17). Glucan endo-1,3-beta-glucosidase and UDP-glucuronosyltransferase were differentially expressed in this study; they thus seem to be closely associated with cold resistance of poplars.

Endoglucanase and exocellobiohydrolase can degrade cellulose into cellobiose, which is then converted to glucose by β-glucosidase. For the effective degradation of cellulose, β-glucosidase is thus pivotal.

UDP-glucuronosyltransferase (UGT, EC 2.4.1.17), an endoplasmic reticulum membrane protein, plays a catalytic role in the transfer of glucuronic acid from UDP-glucuronic acid to hydrophobic molecules. Most UGTs are located in the cytoplasm [35, 36]; in addition, according to some studies, they are located in the endoplasmic reticulum membrane cavity [37] and vacuoles [38], or anchored to the plasma membrane [39] and endoplasmic reticulum membrane [40].

In plants, there are numerous substrates for glycosidation, which is catalyzed by UGT, including plant hormones and secondary metabolites [41]. Under the action of UDP-glycosyltransferase, ABA in plants can exist in the endoplasmic reticulum and vacuoles in the inactive form of glycosides. When plants require ABA, this inactive form is hydrolyzed by the β-glucosidase homologs AtBG1 and AtBG2 to produce ABA [42, 43].

### Relationship between DEGs and DNA repair

**Fig. 10:**
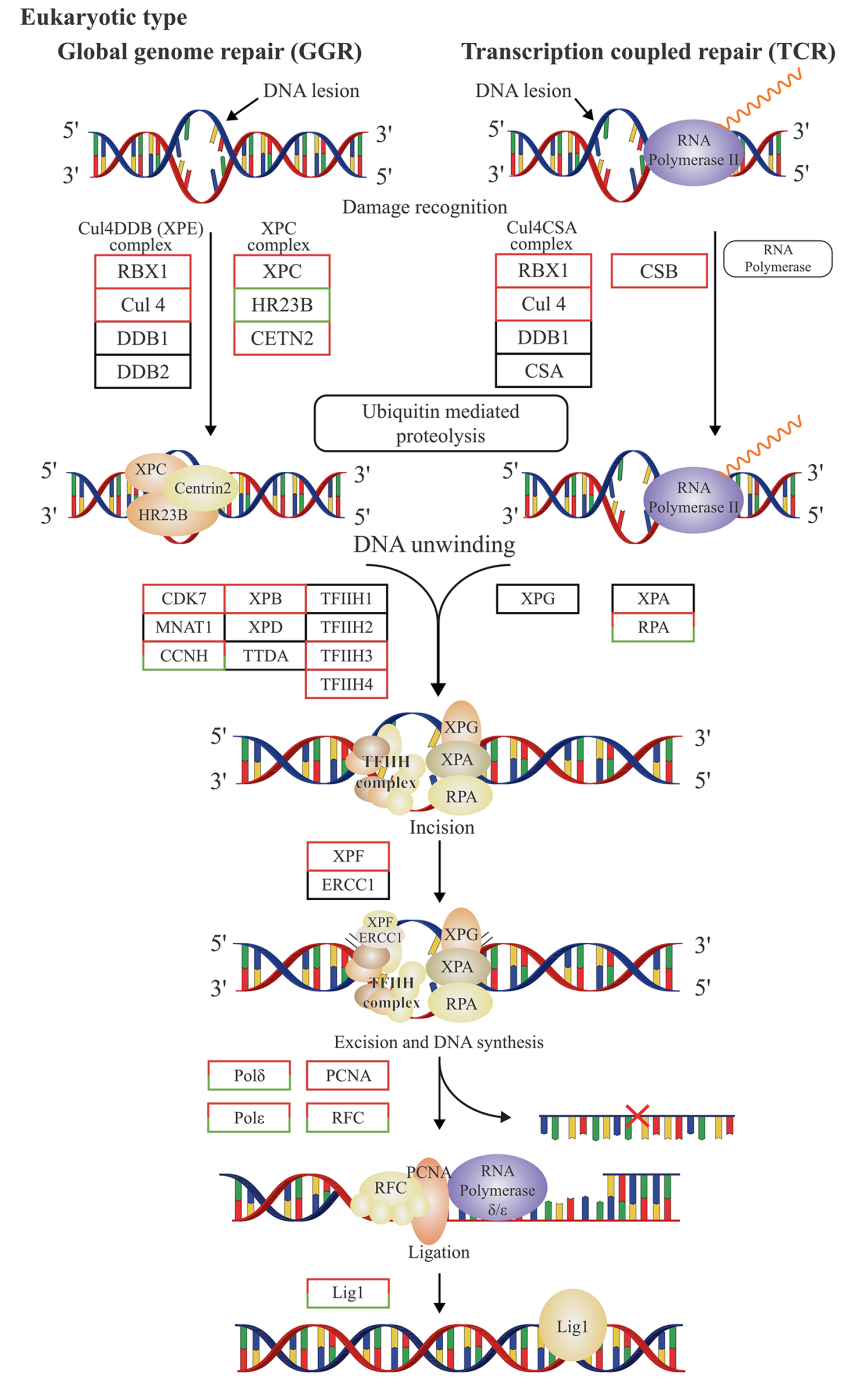
Gene repair pathway in eukaryotes

One of the basic requirements of poplar, a perennial woody plant, is to survive cold temperatures. Its capability to reversibly repair freeze–thaw injury is the key to its survival in repeated freeze–thaw environments. In poplar, the key genes involved in DNA repair play an important role in the repair of freeze–thaw injury. The global genome repair process mainly includes damage recognition, DNA unwinding, excision, and DNA synthesis. Figure 10 shows the gene repair pathway in eukaryotes.

Nucleotide excision repair is one of the repair pathways employed by plants to protect their genome. Xeroderma pigmentosum group B (XPB) helicase (also known as Ercc3/RepB/XPB/Rad25/Ssl2/haywire) is a core subunit of the eukaryotic basal transcription factor complex TFIIH which plays a dual role in transcription and DNA repair [44]. XPB was found to be a single copy gene in eukaryotes, but found as a tandem duplication in the plant *Arabidopsis thaliana*, AtXPB1 and AtXPB2. Results [45] suggest a functional specialization for the AtXPB paralogs: while the AtXPB2 paralog may have a role in cell proliferation and repair as for XPB in other eukaryotes, Raikwar et al. [46] verified a repair function of helicase XPB2 when tobacco was subjected to abiotic stress, including cold stress. DNA repair has not been studied in trees.

Potri.014G050200.v3.0 from the pathway of DNA repair and recombination protein RAD54 and RAD54-like protein (K10875, EC 3.6.4.-) and Potri.001G101300.v3.0 from the pathway of DNA excision repair protein ERCC-3 (K10843, EC 3.6.4.12) were significantly induced at − 40 °C, suggesting their importance in DNA repair. By analysis using the NCBI BLAST tool (https://blast.ncbi.nlm.nih.gov/Blast.cgi), it was found that protein Potri.001G101300.v3.0 had the highest similarity with protein PdeHOE87 (KAH8522005.1; 95.64% sequence similarity). The online subcellular positioning tool Cell-PLoc-2 (www.csbio.sjtu.edu.cn/bioinf/Cell-PLoc-2) predicted that this protein is located in the nucleus. DNA repair genes will be the focus of molecular mechanism analysis of poplar resistance to freeze–thaw stress in the future.

## Conclusion

Poplar is an economically and ecologically important plant species. Low temperatures are known to restrict its growth and geographical distribution. Herein we analyzed the transcriptome of the Euramerican poplar Zhongliao1 to elucidate molecular mechanisms underlying cold stress response. A total of 29,060 genes were detected; furthermore, several key genes related to low temperature stress were identified, which were involved in diverse pathways, including Ca^2+^ and ABA signaling pathways, starch–sucrose metabolism pathway, and DNA repair. Our findings enhance our understanding of the gene expression pattern of Zhongliao1 at normal and low temperatures, and also lay a foundation for cold tolerance breeding.

## Data Availability

The datasets used and/or analyzed during the current study are available from the in the US National Library of Medicine, https://www.ncbi.nlm.nih.gov/bioproject/PRJNA891633.

## Abbreviations

CaM: Calmodulin
CML: Calmodulin-like
DEGs: Differentially expressed genes
MDA: Malondialdehyde
REC: Relative electric conductivity
UGT: UDP-glucuronosyltransferase
XPB: Xeroderma pigmentosum group B
